# Hybrid Cell Structure for Wideband CMUT: Design Method and Characteristic Analysis

**DOI:** 10.3390/mi12101180

**Published:** 2021-09-29

**Authors:** Hongliang Wang, Xiao Huang, Lijun Yu, Qi Ding, Hanqiang Zhang, Changde He, Wendong Zhang

**Affiliations:** National Key Laboratory for Electronic Measurement Technology, Key Laboratory of Instrumentation Science & Dynamic Measurement (North University of China), Ministry of Education, North University of China, Taiyuan 030051, China; huangxiao.nuc.edu@outlook.com (X.H.); weilijun1997@foxmail.com (L.Y.); S1906184@st.nuc.edu.cn (Q.D.); zhqhmz@foxmail.com (H.Z.); hechangde@nuc.edu.cn (C.H.); wdzhang@nuc.edu.cn (W.Z.)

**Keywords:** wideband CMUT, hybrid cell structure, characteristic analysis

## Abstract

Capacitive micromachined ultrasonic transducer (CMUT) is an ultrasonic transducer based on the microelectromechanical system (MEMS). Wideband CMUT has good application prospects in ultrasonic imaging, ultrasonic identification, flow measurement, and nondestructive testing due to its excellent characteristics. This paper studies the method of increasing the bandwidth of the CMUT, proposes the structure of the wideband CMUT with a hybrid cell structure, and analyzes the design principles and characteristics of the wideband CMUT structure. By changing the cell spacing and the number of cells of different sizes composing the CMUT, we analyze the simulation of the effect of the spacing and number on the CMUT bandwidth, thereby optimizing the bandwidth characteristics of the CMUT. Next, the selection principle of the main structural parameters of the wideband CMUT is analyzed. According to the proposed principle, the CMUT in the air and water are designed and simulated. The results prove that both the air and water CMUT meet the design requirements. The design rules obtained in this paper can provide theoretical guidance for the selection of the main structural parameters of the wideband CMUT.

## 1. Introduction

Ultrasound has the characteristics of high frequency, concentrated energy, strong penetrating power, and good directionality [1], and is widely used in ultrasonic imaging, medical diagnosis, non-destructive testing, liquid flow measurement, fault location, biochemical gas detection, and other fields. Among them, in the application of ultrasound imaging, the ultrasound transducer characteristics directly determines the imaging resolution and detection depth of the imaging system [2]. The higher the resonance frequency of the transducer, the higher its imaging resolution, and the easier it is to obtain high-quality images. However, as the resonance frequency increases, its detection depth will decrease, which limits the imaging range of the imaging system. Therefore, imaging resolution and detection depth are a pair of mutually restrictive factors. In order to balance the contradiction between the two as much as possible, the frequency range of the ultrasound transducer should be broadened so that the imaging resolution and detection depth exhibit good performance in this frequency range, thereby improving the imaging quality of the ultrasound imaging system. Wideband ultrasound transducers can not only be used in the field of ultrasound imaging to improve the quality of imaging, but also present unique advantages in many other applications. In flow measurement or ranging applications, the transmission time of ultrasonic pulses needs to be measured [3], and the use of a wideband ultrasonic transducer can improve the accuracy of the transmission time measurement. In ultrasonic identification applications, a wide bandwidth ultrasonic transducer can reduce the pulse width and improve the axial resolution. In short, the research and design of wideband ultrasonic transducers have important practical significance.

Since the 20th century, microelectromechanical systems (MEMS) technology has been rapidly developed. At the same time, a new type of ultrasonic transducer—capacitive micromachined ultrasonic transducer (CMUT)—has also been developed. It gradually replaces piezoelectric ultrasonic transducers and becomes a new generation mainstream ultrasonic transducer by virtue of its advantages in technology, such as easy processing and suitability for mass production.

Compared with piezoelectric ultrasonic transducers, CMUT has significant advantages in structural design, process manufacturing, and integration with front-end signal circuits. First of all, CMUT does not need to add an impedance matching layer [4,5], and it has a larger bandwidth and sensitivity. Secondly, CMUT has a large frequency range. In addition, the CMUT can be integrated with the signal conditioning circuit to form a chip [6], which reduces the influence of external interference signals introduced during the module connection and the parasitic capacitance between the signal conditioning circuits and reduces the energy conversion. The volume of the device realizes the miniaturized design of the ultrasound imaging system [7]. In addition, CMUT has the advantages of simple manufacturing process and low manufacturing cost. Based on the above advantages, CMUT has gradually replaced piezoelectric ultrasonic transducers as the mainstay of wideband ultrasonic imaging transducers. At the same time, wideband CMUT structure design and performance optimization have become a research hotspot for scholars.

Experts and scholars from various countries have proposed various methods to broaden the frequency band of the CMUT and manufacture a wideband ultrasonic transducer with good performance. Hall et al. [8] optimized the mass distribution of the membrane by loading a patterned membrane on a uniform rectangular silicon nitride membrane and obtained a wideband response. On this basis, they designed a two-electrode structure, depositing electrodes on both sides and the center of the membrane, respectively, which enhanced the reception of harmonic signals and further broadened the frequency band of the transmission signal. Bayram et al. [9] optimized the CMUT cell by adjusting the membrane thickness, membrane radius, electrode radius, and cavity height to obtain the largest gain bandwidth product. On this basis, two and three kinds of cells of different sizes are arranged to form an array element, which further improves the bandwidth of the CMUT. Olcum et al. [10] studied the method of increasing the bandwidth of a single CMUT cell and CMUT array in the air domain. First, establish the Mason equivalent circuit model to simulate the equivalent circuit of the CMUT cell. The results show that when the characteristic frequency is fixed, the larger the radius–thickness ratio of the CMUT cell vibration membrane, the wider the bandwidth. In addition, in order to increase the bandwidth of the CMUT array, three CMUT cells with different center frequencies are combined to form an interleaved CMUT array, which achieves a relative bandwidth of more than 60% in the air domain. Manzanares et al. [11] proposed a method of designing an air-coupled CMUT using the principle of Helmholtz resonators, using a resonant cavity with holes instead of a traditional vacuum sealed cavity to increase the output pressure and bandwidth of the CMUT. The finite element simulation results show that the designed CMUT can increase the output pressure and bandwidth in the air. Apte et al. [12] proposed a CMUT structure with vent holes in the cavity. By reasonably selecting the size, number, and location of the vent holes, the squeezing effect can be controlled, thereby increasing the bandwidth of the CMUT. They designed two CMUT devices with a vent area ratio of 0.05% and 1% and performed finite element simulation and actual testing. The results showed that the relative bandwidth of the two CMUT devices in the air domain was 19% and 36%, respectively. Adelegan et al. [13] designed a ring-shaped and spiral-shaped air-coupled CMUT. They first used ANSYS^®^ 16.1 (Canonsburg, PA, USA) for simulation analysis, and then used anodic bonding to make a prototype for testing. Through simulation and testing, they showed that the designed air-coupled CMUT has a relative bandwidth of 12% in the air domain of −6 dB. Based on silicon-on-insulator (SOI) wafer bonding technology, Zhang et al. [14] designed and manufactured a 16-cell air-coupled CMUT array. All cells use circular membranes of the same size, which broadens the bandwidth of the CMUT array by reducing the quality of the membrane. Tests show that the designed air-coupled CMUT array has a resonance frequency of 215 KHz, and its −3 dB relative bandwidth in the air domain has reached 15.7%.

Chee et al. [15,16] used the staggered arrangement of membranes with radius of 82 μm and 36 μm to fabricate a multi-frequency ultrasonic transducer. Tests show that the working frequency of the ultrasonic transducer in the water is from 0.6 to 7.32 MHz, and the relative bandwidth of −6 dB reaches 170%. Compared with an ultrasonic transducer composed of a uniform thin membrane, its bandwidth is increased by 40%. Pun et al. [17] proposed a new single-chip multi-band CMUT structure design. Five CMUT arrays with different sizes are manufactured on the same chip, and the resonances of the CMUT arrays used are concentrated on different frequencies. They manufactured the CMUT and performed simulation tests. The results showed that the −6 dB absolute bandwidth of the designed multi-band CMUT in the water area was 8.8 MHz, and its relative bandwidth reached 140%.

This article mainly focuses on how to increase the bandwidth of the CMUT. First, it introduces the wideband CMUT cells and the influencing factors of the CMUT bandwidth, and then proposes the design method of the wideband CMUT and uses the COMSOL finite element simulation software for modeling. Based on analysis, a CMUT structure with −6 dB relative bandwidths of 50% and 80% in the air and water domains was designed.

## 2. CMUT Frequency Band Impact Analysis

### 2.1. CMUT Basic Structure and Its Main Parameters

**Figure 1:**
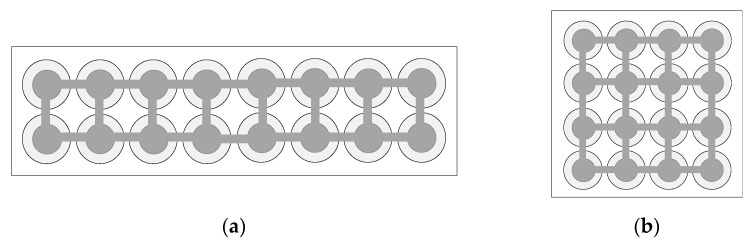
Capacitive micromachined ultrasonic transducer (CMUT) diagram. (**a**) Rectangular diagram; (**b**) Square diagram.

**Figure 2:**
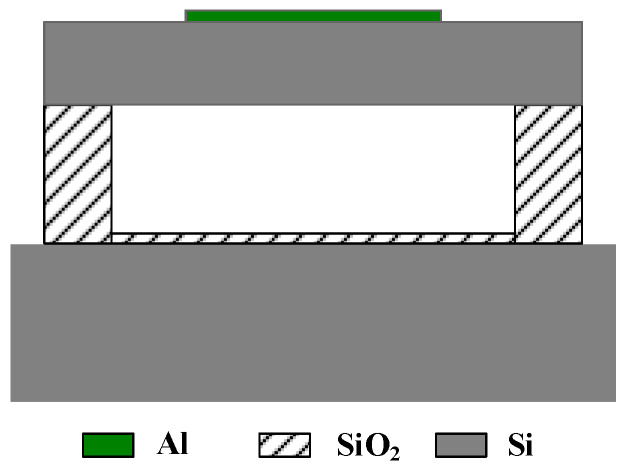
Schematic diagram of CMUT cell cross-section.

Generally, CMUT is composed of many cells arranged and combined according to a certain rule to achieve the requirements of strong directivity, high sensitivity, and high transmission power [18,19]. CMUT can be designed as a rectangle and a square, as shown in Figure 1. Among them, a cross-sectional schematic diagram of the basic unit of CMUT-CMUT cell is shown in Figure 2. It is generally composed of a metal upper electrode, a vibrating membrane, an edge support column, a sealed vacuum cavity, an insulating layer and a silicon lining doped with boron ions. Its structure is similar to that of a parallel plate capacitor. The upper metal electrode and the silicon substrate doped with boron ions can be equivalent to the upper and lower plates of the capacitor, and the vibration membrane, vacuum cavity, and insulating layer are approximately equivalent to the gap between the upper and lower plates of the capacitor. The diaphragm is usually designed into regular shapes such as round, square, hexagon. On the one hand, the insulating layer can prevent the membrane from collapsing or the cavity is broken down and the upper and lower electrodes contact and cause a short circuit. On the other hand, it can be used as a stop layer for sacrificial layer etching to protect the device from damage [20,21]. The sealed vacuum cavity is selected to prevent the CMUT element from being interfered by the external environment, causing energy loss and affecting the normal operation of the device.

The primary consideration in CMUT design is the resonance frequency. When the frequency of the applied excitation signal is equal to the resonance frequency of the transducer, the vibrating membrane resonates and obtains a larger displacement. At this time, the CMUT obtains a larger emission sound pressure and receiving sensitivity, and the conversion efficiency reaches the best.

The first-order resonance frequency of the circular vibrating membrane CMUT fixed around the periphery is [22]:

(1) $$f_{1}=\frac{0.467h}{a^{2}}\sqrt{\frac{E}{\rho (1-\sigma^{2})}}$$

where *a* is the radius of the vibrating membrane, *h* is the thickness, *ρ* is the density, *E* is the Young’s modulus, and *σ* is the Poisson’s ratio.

### 2.2. CMUT Cell Frequency Band Impact Analysis

The size of the CMUT bandwidth is related to the mechanical quality factor, and its value is the reciprocal of the quality factor [23]. Therefore, the smaller the quality factor, the larger the bandwidth of the CMUT; conversely, the larger the quality factor, the smaller the bandwidth of the CMUT. In the design process, a specific bandwidth can be achieved by adjusting the quality factor.

The quality factor *Q* of CMUT can be expressed as:

(2) $$Q=\frac{\omega_{0}M}{R}$$

among them, *ω*_0_ represents the mechanical resonance angular frequency of the CMUT, *M* represents the equivalent mass of the CMUT, and *R* represents the radiation impedance of the CMUT.

The CMUT mechanical resonance angular frequency *ω*_0_ can be obtained by calculating the resonance frequency *f*_0_. The specific process is as follows:

(3) $$\omega_{0}=2\pi f_{0}=\sqrt{\frac{k}{M}}$$

among them, *k* is the equivalent spring constant of the CMUT.

The equivalent mass *M* of the CMUT is approximately replaced by the equivalent mass *M*_m_ of the vibrating membrane [24], as in Equation (4):

(4) $$M\approx M_{m}=1.88\pi a^{2}\rho h$$

The radiation impedance *R* of the CMUT is composed of two parts: the mechanical impedance of the vibrating membrane *R*_m_ and the acoustic impedance of the medium *R*_r_. When the CMUT works in the air domain, the membrane mechanical impedance *R*_m_ is much greater than the medium acoustic impedance *R*_r_, so its radiation impedance *R* is mainly determined by the membrane mechanical impedance *R*_m_. The mechanical impedance is defined as the ratio of the external pressure on the CMUT diaphragm to its vibration speed [25], where the CMUT diaphragm vibration speed can be calculated by the following equation:

(5) $$\bar{v}=\frac{1}{\pi a^{2}}\int_{0}^{a}\int_{0}^{2\pi }v(r)rd\theta dr=\frac{jp}{h\rho \omega }\left[\frac{2(k_{1}^{2}+k_{2}^{2})J_{1}(k_{1}a)I_{1}(k_{2}a)}{ak_{1}k_{2}(k_{2}J_{0}(k_{1}a)I_{1}(k_{2}a)+k_{1}I_{0}(k_{2}a))J_{1}(k_{1}a))}-1\right]$$

Therefore, the mechanical impedance *R*_m_ of the CMUT vibrating membrane can be expressed as:

(6) $$R_{m}=\frac{p}{\bar{v}}=j\omega \rho h\left[\frac{ak_{1}k_{2}(k_{1}I_{0}(k_{2}a)J_{1}(k_{1}a)+ak_{2}I_{1}(k_{2}a)J_{0}(k_{1}a))}{ak_{1}k_{2}(k_{1}I_{0}(k_{2}a)J_{1}(k_{1}a)+k_{2}I_{1}(k_{2}a)J_{0}(k_{1}a))-2(k_{1}^{2}+k_{2}^{2})J_{1}(k_{1}a)I_{1}(k_{2}a)}\right]$$

In the equation, *ω* represents the angular frequency, *J*_0_(*x*) represents the first-order zero-order Bessel function, *J*_1_(*x*) represents the first-order first-order Bessel function, *I*_0_(*x*) represents the first kind of zero-order modified Bessel function, *I*_1_(*x*) represents the first kind of first-order modified Bessel function, and *k*_1_ and *k*_2_ are respectively given by:

(7) $$\left\{ \begin{array}{l} k_{1}=\sqrt{\frac{\sqrt{d^{2}+4c\omega^{2}}-d}{2c}}\\ k_{2}=j\sqrt{\frac{\sqrt{d^{2}+4c\omega^{2}}+d}{2c}} \end{array}\right.$$

among them:

(8) $$c=\frac{(E+T)h^{2}}{12\rho (1-\delta^{2})}$$

(9) $$d=\frac{T}{\rho }$$

*E* and *T* are the Young’s modulus and residual stresses of CMUT membrane, respectively.

Simplify Equation (3), Equation (4), and Equation (6), respectively, and substitute them into Equation (2) to calculate:

(10) $$\left\{ \begin{array}{l} \omega_{0}\approx \sqrt{\frac{8.5Eh^{2}}{\rho \left(1-\sigma^{2}\right)a^{4}}}\propto \frac{h}{a^{2}}\\ M\approx 1.88\pi a^{2}\rho h\propto a^{2}h\\ R\approx j\omega \rho ah\propto ah\\ Q=\frac{\omega_{0}M}{R}\propto \frac{h}{a} \end{array}\right.$$

It can be seen from the above equation that the quality factor of the CMUT is proportional to the thickness–radius ratio *h/a* of the vibrating membrane. Since the bandwidth of the CMUT is the reciprocal of the quality factor, the bandwidth of the CMUT is proportional to the radius–thickness ratio *a*/*h* of the vibrating membrane. When the resonance frequency of the CMUT is fixed, *a*/*h* should be increased as much as possible to increase the bandwidth of the CMUT cell.

In order to verify the correctness of the above theory, this section will establish a CMUT cell model in the COMSOL finite element software for simulation analysis, and compare the bandwidth of the CMUT cell with different vibrating membrane radius–thickness ratios *a*/*h*.

Observing Equation (1), it can be seen that when the material of the CMUT cell vibration membrane is determined, the equation leaves three variables: CMUT resonance frequency, vibration membrane radius, and vibration membrane thickness. When two of these variables are determined, the other variable is then determined. The resonance frequency is a design index, and its parameters are usually specified before the CMUT design. Therefore, in the design process, when a suitable vibration membrane radius is selected, the thickness of the vibration membrane will be fixed accordingly.

This article is aimed to design a CMUT cell with a resonance frequency of 3 MHz in the air. In order to compare the bandwidth of CMUT cells with different vibrating membrane radius–thickness ratios, the CMUT cell vibrating membrane radius *a* was respectively specified as 60 μm, 120 μm, 240 μm, then calculate according to Equation (1), and finally the vibration that meets the requirements was got. The membrane thickness values *h* are 2.6 μm, 10.4 μm, 41.6 μm, and the corresponding vibration membrane radius–thickness ratio *a*/*h* are 23, 11, and 6, respectively. The other structural parameters are shown in Table 1 and Table 2, and then we used COMSOL Multiphysics 5.4 (COMSOL Inc., Stockholm, Sweden) to perform finite element modeling and analysis.

First, define a constant *V*_dc_ and a sine function *f*(t) as the direct current (DC) bias voltage and alternate current (AC) excitation voltage applied between the upper and lower electrodes of the CMUT cell, respectively. Among them, *V*_dc_ is respectively set as their respective collapse voltage, *f*(t) frequency is 3 MHz, amplitude is 1 V, and lasts for 5 cycles. Secondly, the finite element model of the CMUT cell is established. The size of the vibrating membrane is 60 μm–2.6 μm, 120 μm–10.4 μm, and 240 μm–41.6 μm. Next, the materials were added, and the physics was configured. Here, the physical field chooses electromechanical electric field and pressure acoustics, transient field, the scope of electromechanical electric field is the CMUT cell structure, and the scope of pressure acoustics and transient field is the environmental domain. The applicable boundary of multiphysics was selected, and the software will automatically couple the two physics. Two terminals are added to the electromechanical electric field, the type is set to potential, one of the potential values is set to *V*_dc_ + *f*(t), and the scope is the upper electrode. The other potential value is set to 0, and the scope is selected as the substrate. By setting these two terminal conditions, the purpose of applying a DC bias voltage and an AC excitation voltage between the upper and lower electrodes of the CMUT cell is realized.

**Figure 3:**
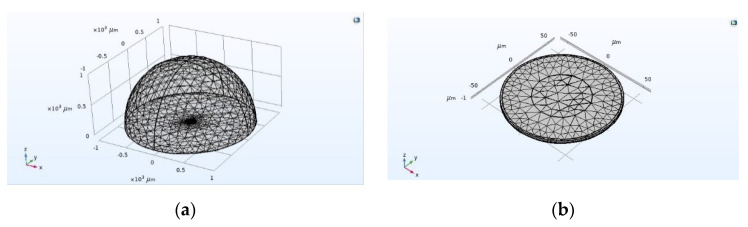
Meshing of CMUT finite element simulation model. (**a**) Meshing of the whole model, (**b**) Meshing of CMUT cell.

**Figure 4:**
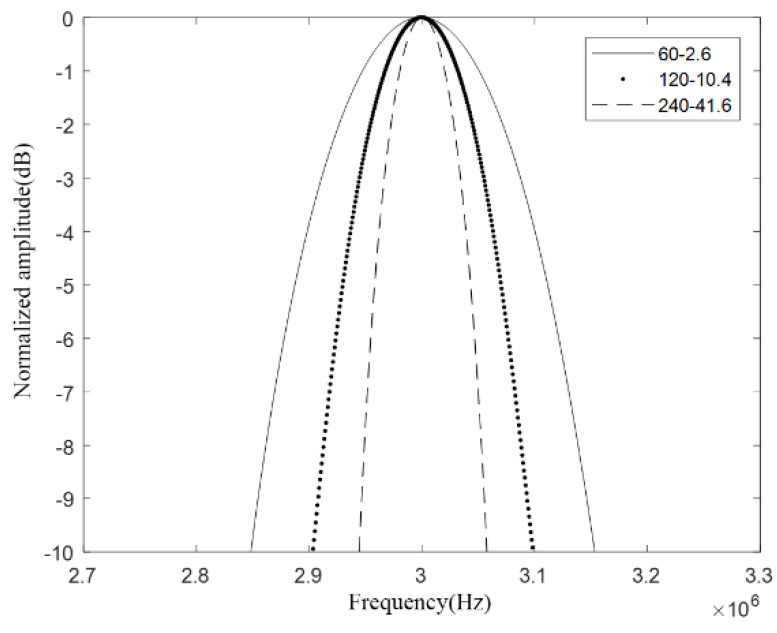
Comparison of CMUT cell bandwidth.

In pressure acoustics, spherical wave radiation is added to the transient field, which acts on the outer boundary of the environmental domain. Spherical wave radiation can absorb the ultrasonic waves emitted by the CMUT, so that errors caused by the reflection of ultrasonic waves at the boundary can be avoided. Next, proceed to meshing and solver settings. Here, the user-controlled grid is selected for meshing. First, the free triangular meshing is performed on the upper surface of the vibrating membrane of the CMUT cell, and then the resulting mesh is scanned, and the scope is selected for the entire CMUT cell part. Finally, the remaining environment domain is divided into unstructured meshes. The completed grid is shown in Figure 3, where Figure 3a is the grid of the entire finite element simulation model, and Figure 3b is the grid of the CMUT cell model. The research type here selects transient analysis, the solution range is 0–20 μs, the step size is 0.1 μs, and the solver selects the transient solver. Finally, the solution is solved. In the result options, one can see the solution results obtained by the finite element analysis. Export the obtained sound pressure data and use MATLAB R2020a (The MathWorks, Inc., Natick, MA, USA) for further analysis and processing. Finally, the CMUT cell bandwidth diagram with different vibrating membrane radius–thickness ratio *a*/*h* as shown in Figure 4 is obtained.

Analyzing the above figure, it can be seen that the radius–thickness ratio of the CMUT cell vibrating membrane will affect the bandwidth of the CMUT cell. Among them, when the radius of the CMUT cell vibration membrane is 60 μm and the thickness is 2.6 μm, the radius–thickness ratio *a*/*h* is 23. At this time, the emission sound pressure bandwidth is the largest, and its −6 dB absolute bandwidth is about 0.3 MHz, and the relative bandwidth reaches 10%. When the radius of the CMUT cell vibration membrane is 120 μm and the thickness is 10.4 μm, the radius–thickness ratio *a*/*h* is 11, and the emission sound pressure bandwidth is in the middle, and its −6 dB absolute bandwidth is about 0.2 MHz, and the relative bandwidth is about 6.7%. When the radius of the CMUT cell vibration membrane is 240 μm and the thickness is 41.6 μm, the radius–thickness ratio *a*/*h* is 6, at this time the emission sound pressure bandwidth is the smallest, and its −6 dB absolute bandwidth is about 0.1 MHz, and the relative bandwidth is only 3.3%.

It can be seen that increasing the radius–thickness ratio *a*/*h* of the vibrating membrane will increase the bandwidth of the CMUT cell. According to Equation (1), it can be seen that the resonance frequency of the circular vibrating membrane CMUT is proportional to the *h*/*a*^2^ of the membrane. If the resonance frequency of the CMUT element is fixed, as the vibration membrane *a*/*h* increases, the radius a of the membrane will decrease, and the decrease of the radius will bring great challenges to the process. Therefore, in the design process, under the premise of meeting the existing technological conditions, the *a*/*h* ratio should be increased as much as possible so as to meet the requirements of the wideband CMUT cell structure design.

### 2.3. CMUT Frequency Band Impact Analysis

**Figure 5:**
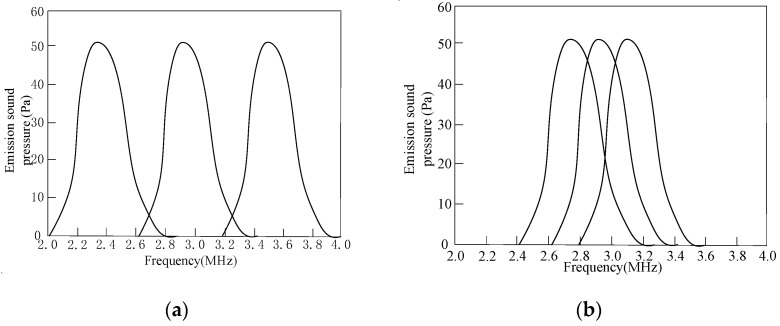
CMUT’s sound pressure bandwidth. (**a**) The frequency range does not overlap, (**b**) Frequency ranges overlap.

In order to improve the characteristics of the CMUT’s emission sound pressure, receiving sensitivity, directivity, bandwidth, etc., multiple CMUT cells can be arranged according to a specific rule to form a CMUT. The composed CMUT can generally be divided into two types, one is uniformly arranged by the same cells, and the other is staggered by different cells. When the CMUT is composed of N identical cells, its emission sound pressure, receiving sensitivity and other parameters will be N times that of a single cell [26]. When the CMUT is composed of N different cells, each cell effectively transmits signals in a small frequency range, and the bandwidth of the overall CMUT will increase. Theoretically, the emission sound pressure bandwidth of a CMUT composed of three different cells is shown in Figure 5. Figure 5a shows a case where the center frequencies of the three types of cells are adjacent, and the frequency ranges do not overlap; Figure 5b shows a case where the center frequencies of the three types of cells are adjacent, and the frequency ranges partially overlap.

It can be seen from Figure 5 that the superposition of the transmitted sound pressure frequency range of the CMUT composed of three different cells can achieve the expected purpose of increasing the bandwidth. However, in Figure 5a, the resonance frequencies of the three types of cells are quite different, so that the main peak of the CMUT cannot be smoothly transitioned, and the obtained frequency range is not flat enough, which affects the quality of the output sound pressure. The three types of cells selected in Figure 5b have adjacent resonance frequencies, and the frequency ranges partially overlap. The problem that the entire CMUT frequency range is not flat enough has been partially resolved.

**Figure 6:**
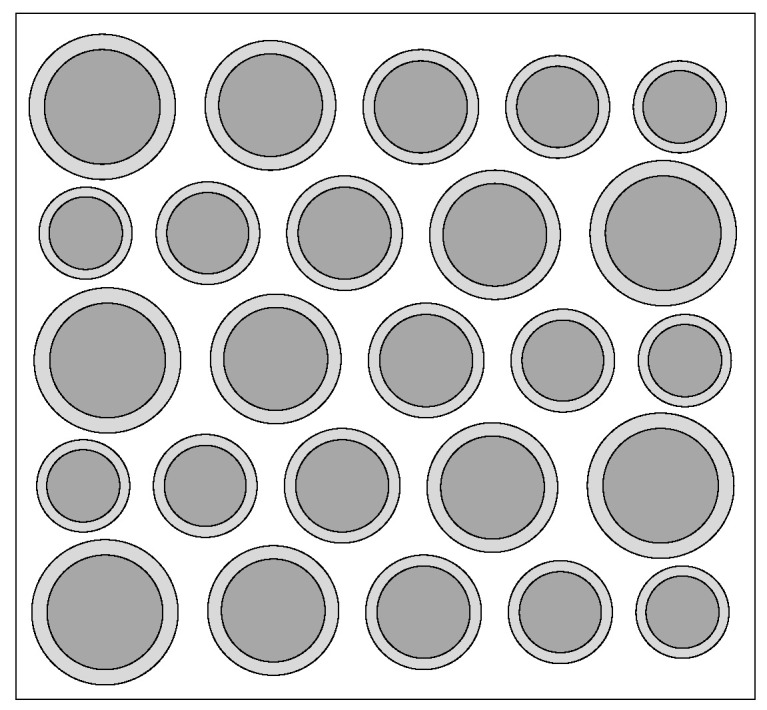
Hybrid cell structure CMUT model diagram.

Based on this principle, this paper designs a CMUT with a hybrid cell structure, as shown in Figure 6. Among them, the cell vibrating membrane that composes the CMUT has the same thickness and successively decreasing radius, that is, the frequency of the CMUT cell increases sequentially, and the frequency range of each cell must overlap while the frequency is increasing. Among the cells that make up the CMUT, the cell whose resonance frequency is the same as that of the CMUT is called the central cell. This cell is the main body of the CMUT design. The frequency range can be adjusted by increasing or decreasing the number of other frequency cells to obtain a suitable bandwidth of the CMUT.

#### 2.3.1. Finite Element Simulation Analysis of Wideband CMUT

**Figure 7:**
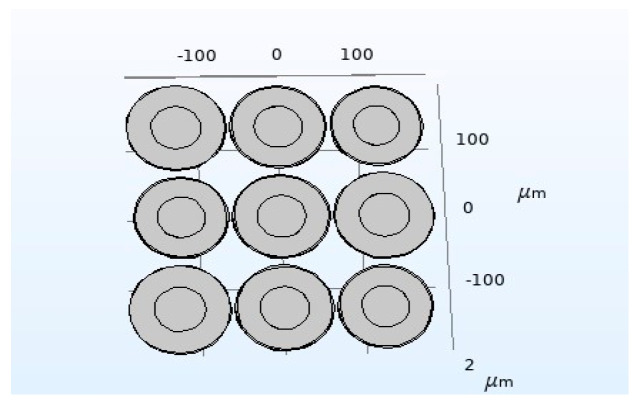
A CMUT model of three cell sizes.

**Figure 8:**
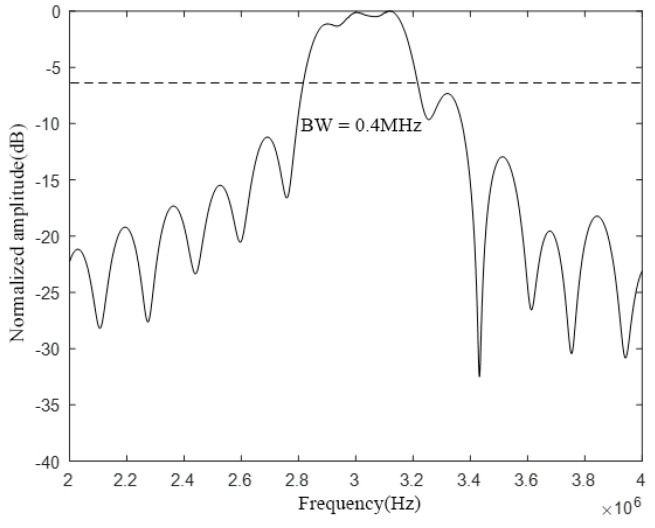
A CMUT bandwidth consisting of three cell sizes.

In this section, a CMUT structure composed of three kinds of cells with vibrating membrane sizes of 59 μm–2.6 μm, 60 μm–2.6 μm, and 61 μm–2.6 μm is established. The resonance frequencies of the three types of cells in the air are 3.13 MHz, 3.02 MHz, and 2.93 MHz, respectively. The structural parameters of them are shown in Table 3, the number of cells is 9, and the spacing is 5 μm. Through COMSOL finite element simulation analysis and MATLAB calculation, the bandwidth of the CMUT in the air domain is obtained. The specific analysis process is as follows:

In COMSOL, the CMUT finite element simulation model as shown in Figure 7 is established, then the materials are added and the physical field conditions is configured, and mesh division is performed, then the corresponding research type and solver is configured, finally post-processing. The overall setting method is the same as that in Section 2.2. Here, the DC bias voltage is selected to correspond to the collapse voltage of the cell with the diaphragm size of 60 μm–2.6 μm, which corresponds to 44 V, and the frequency of the AC excitation voltage is set to 3 MHz. This is because the resonance frequency of the CMUT designed in this section is 3 MHz, and the resonance frequency of the CMUT cell with the diaphragm size of 60 μm–2.6 μm is exactly 3 MHz, so this cell is the central cell. Finally, the bandwidth of the CMUT composed of three different sizes of cell structures is obtained, as shown in Figure 8.

It can be seen from Figure 8 that the bandwidth of the CMUT composed of three different sizes of cell structures has increased to 0.4 MHz, which proves that the designed cell hybrid structure can increase the bandwidth of the CMUT.

#### 2.3.2. Bandwidth Performance Analysis of Wideband CMUT

In this section, we will change the cell spacing of the CMUT and the number of cells of different sizes, analyze the impact of spacing and number on the CMUT bandwidth, and further optimize the bandwidth characteristics of the CMUT.

**Figure 9:**
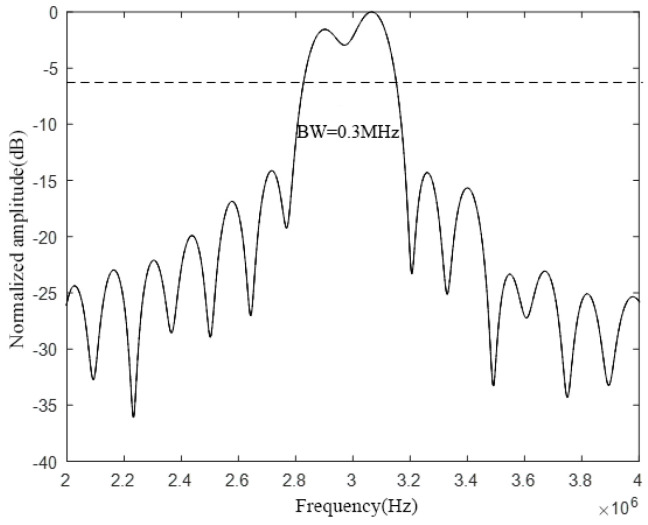
CMUT bandwidth with a cell spacing of 60 μm.

**Figure 10:**
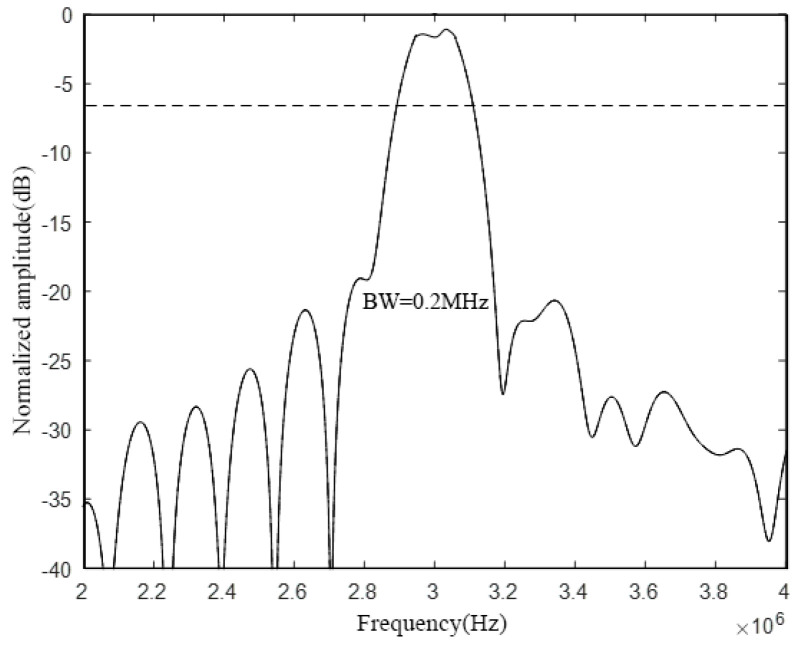
CMUT bandwidth with a cell spacing of 120 μm.

First, the influence of the cell spacing on the bandwidth characteristics of the CMUT is analyzed. Next, the CMUT model composed of three different cell sizes used in the previous section is selected, and the spacing between the cells is set to 60 μm and 120 μm, respectively. Following that, finite element modeling analysis in COMSOL and calculation in MATLAB is performed. Finally, the bandwidth of the CMUT when using different cell spacing is obtained, and the results are shown in Figure 9 and Figure 10, respectively.

Comparing and analyzing Figure 8, Figure 9, and Figure 10, it is found that the change of the cell spacing will affect the bandwidth of the CMUT. When the spacing between CMUT cells is 5 μm, the −6 dB absolute bandwidth of the CMUT is 0.4 MHz; when the spacing is 60 μm, the −6 dB absolute bandwidth of the CMUT is 0.3 MHz; when the spacing is 120 μm, the −6 dB absolute bandwidth of the CMUT is 0.2 MHz. It can be found that the bandwidth of the CMUT decreases with the increase of its cell spacing. This is because with the increase of the cell spacing, the filling factor of the CMUT gradually decreases. When the filling factor is low, each cell vibrating membrane acts more like a separate element, which pushes the medium in the normal direction. The medium is also pushed to the side, and the power quality of the medium increases, resulting in a decrease in the CMUT bandwidth. Therefore, in the CMUT design process, the cell spacing should be reduced as much as possible to increase the bandwidth of the CMUT.

**Figure 11:**
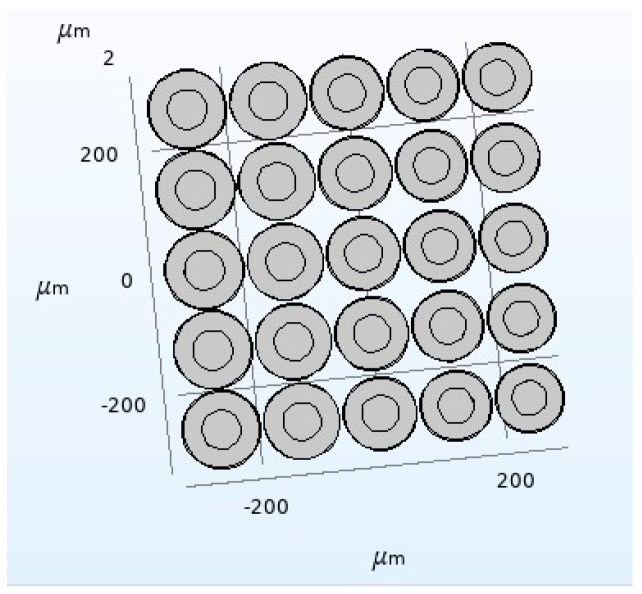
A CMUT model of five cell sizes.

**Figure 12:**
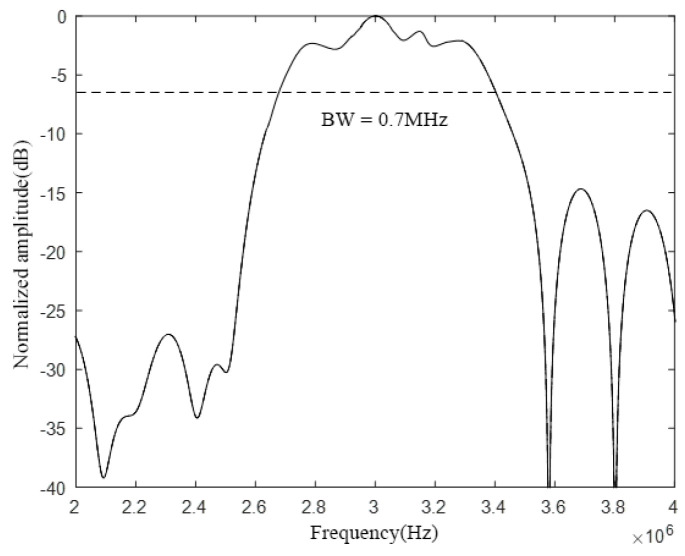
CMUT bandwidth composed of five cell sizes.

Next, a CMUT finite element model composed of five different cell sizes is constructed. The radius of the cell vibrating membrane composing the CMUT are 58 μm, 59 μm, 60 μm, 61 μm, and 62 μm, and the thickness of the membrane is 2.6 μm. The rest of the structural parameters are shown in the Table 4 and the corresponding resonance frequencies in the air are 3.24 MHz, 3.13 MHz, 3.02 MHz, 2.93 MHz, and 2.83 MHz, respectively. By comparing the bandwidth characteristics of a CMUT composed of three different cell sizes and a CMUT composed of five different cell sizes, the influence of the number of cells of different sizes on the bandwidth characteristics of the CMUT is finally analyzed. The specific analysis process of the bandwidth characteristics of the CMUT composed of five different sizes of cells is as follows:

A CMUT finite element simulation model composed of five vibrating membrane sizes in COMSOL is established as shown in Figure 11. The structure is composed of 25 cells with a spacing of 5 μm between each cell, and materials to configure the physical field conditions is added, then meshing, and the corresponding research type and solver is configured, then post-processing. The setting method is the same as the method presented in Section 2.2. The DC bias voltage is set to 44 V, and the frequency of the AC excitation voltage is set to 3 MHz. Finally, the bandwidth of the CMUT composed of five different sizes of cell structures, as shown in Figure 12, is obtained.

Comparing Figure 8 and Figure 12, it can be found that the number of cells of different sizes will affect the bandwidth characteristics of the CMUT. When the CMUT is composed of three different sizes of cells, its −6 dB absolute bandwidth is 0.4 MHz (13.3%); when it is composed of five different sizes of cells, the −6 dB absolute bandwidth of the CMUT is 0.7 MHz (23.3%). It can be seen from this that when the number of cells of different sizes that make up the CMUT increases, its bandwidth becomes wider. Therefore, in order to increase the bandwidth of the CMUT, the CMUT should be composed of a variety of cells of different sizes.

## 3. Wideband CMUT Design Method

From the above analysis, it can be seen that the CMUT of the cell hybrid structure can achieve the purpose of increasing the bandwidth. The number of cells of different sizes and the size of each cell are the main structural parameters of the CMUT, which directly affect the resonance frequency and bandwidth of the CMUT. Therefore, in the CMUT design process, the appropriate number and size of the cell should be selected. Among them, the cell size can be described by the thickness and radius of the vibrating membrane. On the one hand, the thickness and radius of the vibrating membrane determine the resonance frequency of the cell; on the other hand, when the thickness and radius of the membrane are determined, the radius of structures such as electrodes and cavities is determined accordingly. This section will mainly consider when the CMUT resonance frequency and the relative bandwidth of −6 dB are determined, the number of cells of different sizes, and the selection principle of the thickness and radius of the vibrating membrane of each cell are to be defined.

**Figure 13:**
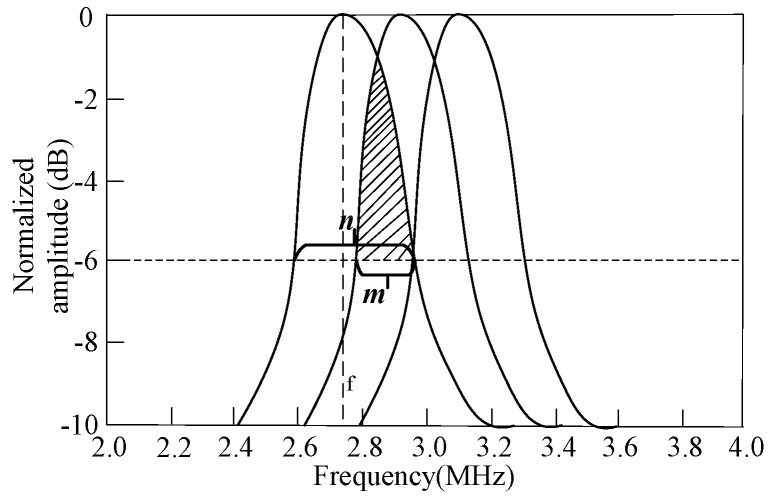
CMUT made up of different cell sizes.

From the analysis in Section 2.3, it can be seen that when the frequency ranges of adjacent cells overlap, the CMUT has better bandwidth characteristics. As shown in Figure 13, the ratio of the frequency *m* of the overlapping part of two adjacent cells to the absolute bandwidth *n* of the cell is recorded as the frequency cross-over range of the adjacent cells. Since the absolute frequencies of the adjacent two cells are similar, the absolute frequency of any cell can be selected here. The ratio of the −6 dB absolute bandwidth *n* of the cell to its resonance frequency *f* is recorded as the −6 dB relative bandwidth. Assuming that the frequency crossover range is *w* (= *m*/*n*), the −6 dB relative bandwidth of each cell is *x*, and the number of cells of different sizes is *N*, the −6 dB relative bandwidth *y* of the composed CMUT can be expressed as:

(11) y=x+(N−1)(1−w)x

Analyzing the above equation, it can be found that when the frequency range of adjacent cells forming a CMUT is determined, only the −6 dB relative bandwidth of the cell and the number of cells of different sizes are needed to calculate the −6 dB relative bandwidth of the CMUT. Conversely, when the −6 dB relative bandwidth of the CMUT and CMUT is known, the number *N* of different sizes required can be calculated, as shown in the following equation:

(12) $$N=\frac{\left(y-x\right)}{(1-w)x}+1$$

Generally, the −6 dB relative bandwidth *x* of the CMUT cell can be known based on previous experience, and the −6 dB relative bandwidth *y* of the CMUT will be given as an indicator before the design. In order to be able to use the resonance frequency of the central cell as the resonance frequency of the CMUT, the number of cells on the left and right sides of the central cell should be the same, so the number *N* of cells of different sizes should be an odd number. When the calculation result *N* is an odd number, it is sufficient to select *N* cells, and when the calculation result *N* is an even number, *N* + 1 cells should be selected.

The central cell of the CMUT is recorded as the 0th cell, the cell adjacent to the central cell is recorded as the first cell, and the cell adjacent to the first cell is recorded as the second cell. By analogy, the cell located at the edge is recorded as the *K*th cell. Among them, the resonance frequency of the *i*-th element is calculated by the following Equation:

(13) $$f_{i}=f_{0}\pm (1-w)\sum_{0}^{K-1}BW_{i}$$

*f*_0_ is the resonance frequency of the CMUT, and *BW_i_* is the absolute bandwidth of the *i*-th infinite element. When the cell is on the left side of the center cell, the symbol is “+”, and when the cell is on the right, the symbol is “−”.

Because the resonance frequency of the central cell is the same as the resonance frequency of the CMUT. Therefore, when the resonant frequency and bandwidth of the CMUT are given, the resonant frequency of the center cell is determined accordingly, and then the radius and thickness of the center cell vibrating membrane can be calculated according to Equation (1). In this process, a smaller vibration membrane radius should be selected according to the technological level of the laboratory, so as to increase the radius–thickness ratio of the CMUT vibration membrane and obtain a wide bandwidth CMUT cell. The thickness of the vibrating membrane of the cell constituting the CMUT is the same, so when the thickness of the vibrating membrane of the central cell is determined, the thickness of the vibrating membrane of the other cell is determined accordingly. In addition, the resonance frequency of the *i*-th infinite element can be calculated according to Equation (13), and the obtained frequency and the thickness of the vibrating membrane can be substituted into Equation (1) to obtain the radius of the *i*-th infinite element vibrating membrane. So far, the number of cells of different sizes constituting the CMUT and the vibration membrane parameters of each cell can be obtained.

## 4. Design and Analysis of Wideband CMUT

### 4.1. Air Domain Wideband CMUT Structural Design and Finite Element Analysis

According to the previous research of the research group, a CMUT cell with a relative bandwidth of about 10% in the air domain can be designed with a −6 dB relative bandwidth. On this basis, this paper designs a CMUT with a resonance frequency of 3 MHz in the air and a −6 dB relative bandwidth of not less than 50%. This verifies the correctness of the above Equation and analyzes the influence of the adjacent cell frequency cross range *w* on the CMUT bandwidth characteristics. Here, 20%, 50%, and 80% are selected for *w*, respectively. Through calculation, the number of different size cells required is shown in Table 5.

When *w* is 20%, the required number *N* of cells of different sizes is calculated by Equation (12) to be an even number 6. At this time, the number of cells should be *N* + 1, so at least 7 different sizes of cells should be selected for combination to meet the design requirements. According to Equation (13), the resonance frequency of each kind of cell can be calculated, respectively: 2.33 MHz, 2.53 MHz, 2.86 MHz, 3 MHz, 3.24 MHz, 3.5 MHz, 3.78 MHz.

**Figure 14:**
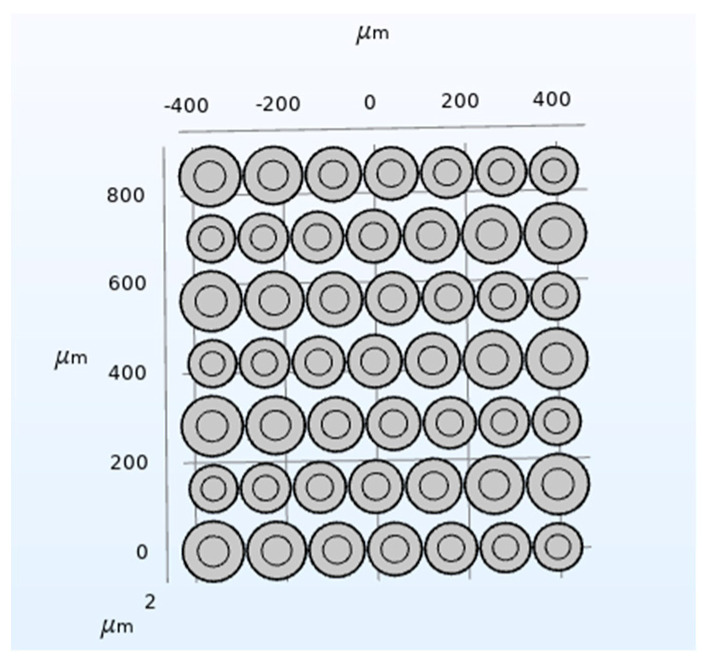
CMUT structure (*w* = 20%).

**Figure 15:**
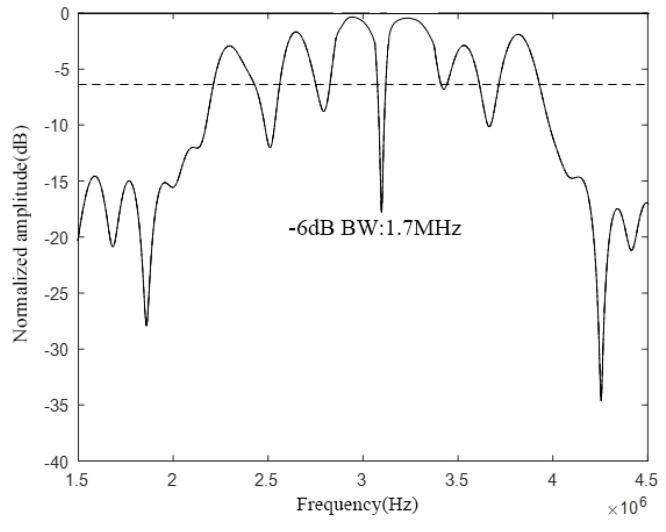
CMUT bandwidth (*w* = 20%).

According to the above analysis, the CMUT structure shown in Figure 14 is designed, and then the COMSOL finite element simulation software is used for modeling and analysis, and finally the bandwidth of the CMUT is shown in Figure 15.

It can be seen from Figure 15 that when *w* is 20%, the resonant frequency of the designed CMUT in the air domain is 3 MHz, the −6 dB absolute bandwidth is 1.7 MHz, and its relative bandwidth is 56.7%, which meets the design requirements. However, in the −6 dB frequency range, the curve fluctuates greatly, which is caused by the small frequency crossover range between each cell.

When *w* is 50%, it can be calculated according to Equations (12) and (13) that at least 9 kinds of cells with different frequencies are required to meet the design requirements. Among them, the resonance frequency of each cell is: 2.44 MHz, 2.57 MHz, 2.7 MHz, 2.85 MHz, 3 MHz, 3.15 MHz, 3.3 MHz, 3.47 MHz, 3.65 MHz.

**Figure 16:**
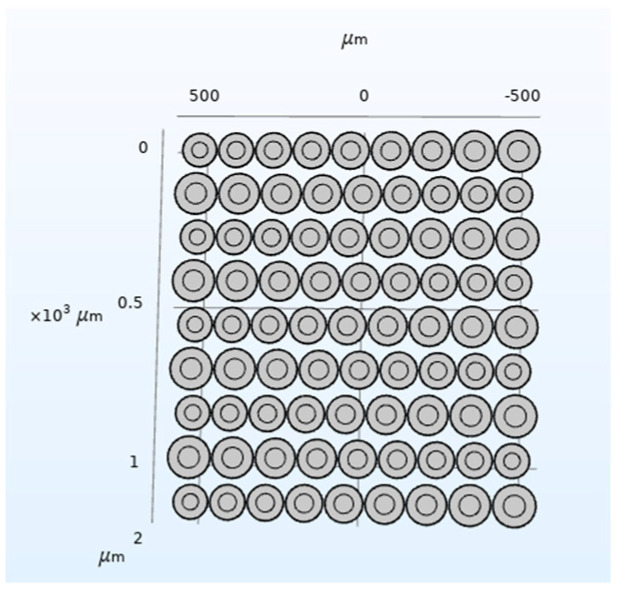
CMUT structure (*w* = 50%).

**Figure 17:**
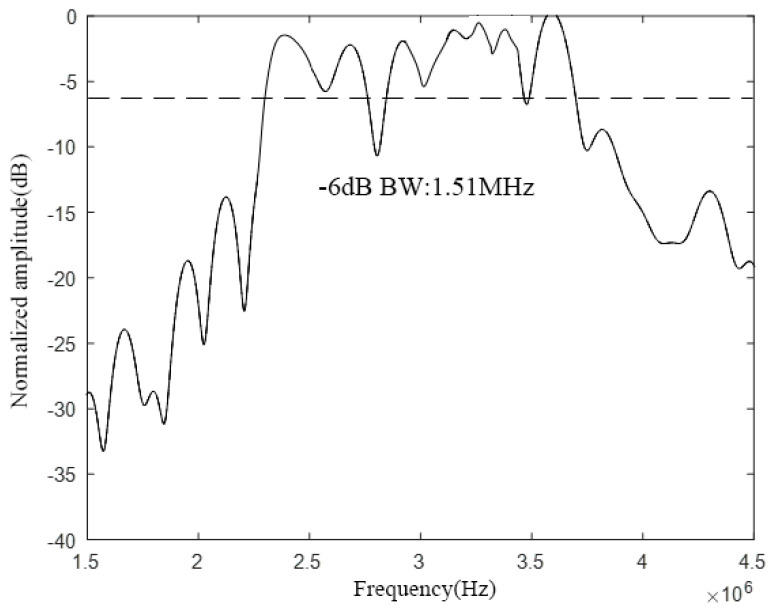
CMUT bandwidth (*w* = 50%).

Next, the CMUT structure model was constructed in COMSOL, as shown in Figure 16. Through finite element analysis, the bandwidth of the CMUT, as shown in Figure 17, is finally obtained.

It can be seen from Figure 17 that when *w* is 50%, the designed CMUT’s resonance frequency in the air domain is 3 MHz, the −6 dB absolute bandwidth is 1.51 MHz, and its relative bandwidth is 50.3%, which meets the design requirements. In addition, in the frequency range of −6 dB, the curve has two frequencies with large fluctuations, exceeding 5 dB, and the rest is relatively flat.

When *w* is 80%, it can be calculated according to the Equation that at least 21 kinds of cells with different frequencies are required to meet the design requirements. Among them, the resonance frequency of each cell is 2.45 MHz, 2.5 MHz, 2.55 MHz, 2.6 MHz, 2.66 MHz, 2.71 MHz, 2.77 MHz, 2.82 MHz, 2.88 MHz, 2.94 MHz, 3 MHz, 3.06 MHz, 3.12 MHz, 3.18 MHz, 3.24 MHz, 3.31 MHz, 3.38 MHz, 3.44 MHz, 3.51 MHz, 3.58 MHz, 3.66 MHz.

**Figure 18:**
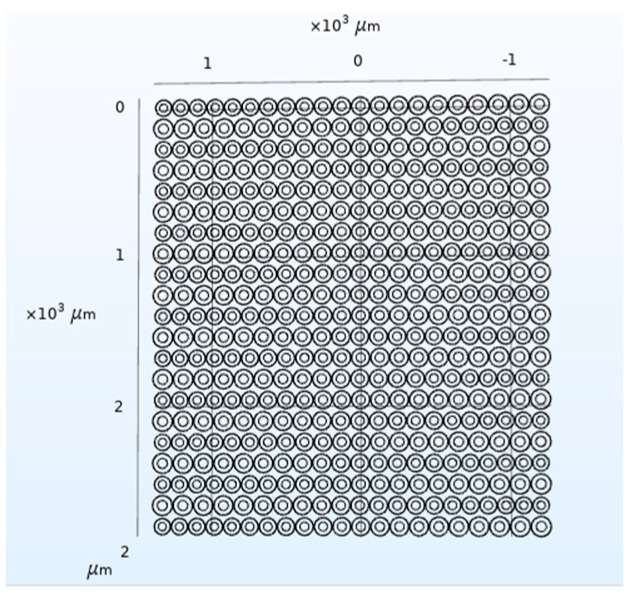
CMUT structure (*w* = 80%).

**Figure 19:**
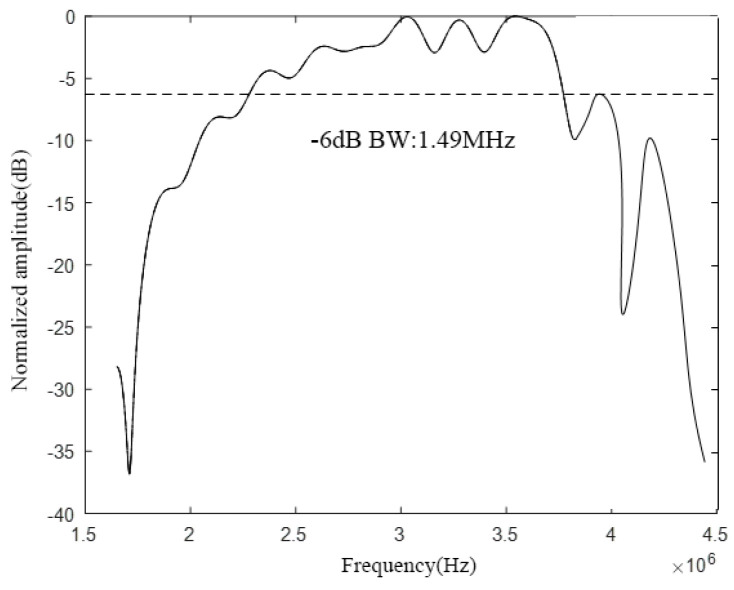
CMUT bandwidth (*w* = 80%).

According to the above analysis, the CMUT structure shown in Figure 18 is designed, and then the COMSOL finite element simulation software is used for modeling and analysis, and finally the bandwidth of the CMUT is shown in Figure 19.

It can be seen from Figure 19 that when *w* is 80%, the designed CMUT’s resonance frequency in the air domain is 3 MHz, the −6 dB absolute bandwidth is 1.49 MHz, and its relative bandwidth is 49.7%, which roughly meets the design requirements. In addition, in the frequency range of −6 dB, the curve has very small fluctuations, none of which exceed 5 dB, and the overall curve is relatively flat.

It can be seen from the above analysis that when *w* is 20%, 50%, and 80%, the CMUT designed according to Equations (12) and (13) achieves a resonance frequency of 3MHz in the air and a relative bandwidth of −6 dB of 50%. This shows that the principle of selecting the main structural parameters of the wideband CMUT proposed in this article is feasible and can be used as a theoretical basis for the design of the wideband CMUT structure. When *w* is 20%, the CMUT frequency curve is not flat in the range of −6 dB, and the fluctuation is large, which is not conducive to the normal operation of the wideband CMUT; when *w* is 50%, the frequency curve is much flat, with only two frequencies The fluctuation exceeds 6 dB; when *w* is 80%, the CMUT frequency curve is the flattest, and the fluctuation does not exceed 5 dB, which satisfies the requirement of a wideband CMUT. It can be seen that, when the frequency crossover range between adjacent cells is larger, the CMUT frequency curve formed is flatter and the bandwidth characteristic is better. Therefore, in the process of designing a wideband CMUT, the frequency crossover range between adjacent cells should be as large as possible, usually greater than 50%, so as to obtain better bandwidth characteristics.

### 4.2. Waters Domain Wideband CMUT Structural Design and Finite Element Analysis

According to the previous research of the research group, it is possible to design a CMUT cell with a relative bandwidth of about 20% in the water area of −6 dB. On this basis, this paper designs a CMUT with a resonance frequency of 1.1 MHz in the water and a −6 dB relative bandwidth of not less than 80% to further verify the correctness of the above Equation. From the previous analysis, we can see that the larger the frequency crossover range between adjacent cells, the flatter the formed CMUT frequency curve and the better the bandwidth characteristic. Therefore, 80% is selected here.

It is calculated by Equation (12) that the required number *N* of cells of different sizes is an even number 16. At this time, the number of cells should be selected as *N* + 1, so at least 17 kinds of cells of different sizes are selected for combination to meet the design requirements. According to Equation (13), the resonance frequency of each kind of cell can be calculated, which are 0.8 MHz, 0.83 MHz, 0.86 MHz, 0.9 MHz, 0.94 MHz, 0.98 MHz, 1.02 MHz, 1.06 MHz, 1.1 MHz, 1.14 MHz, 1.18 MHz, 1.23 MHz, 1.28 MHz, 1.33 MHz, 1.38 MHz, 1.44 MHz, 1.50 MHz.

**Figure 20:**
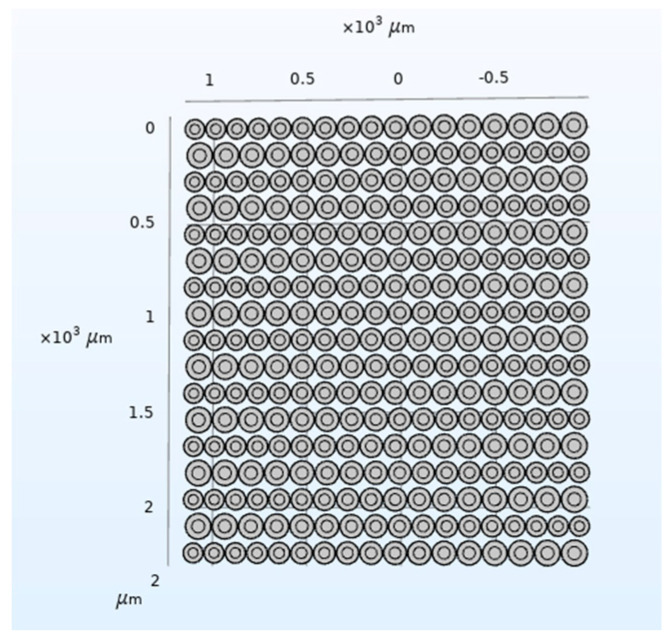
CMUT structure in waters.

**Figure 21:**
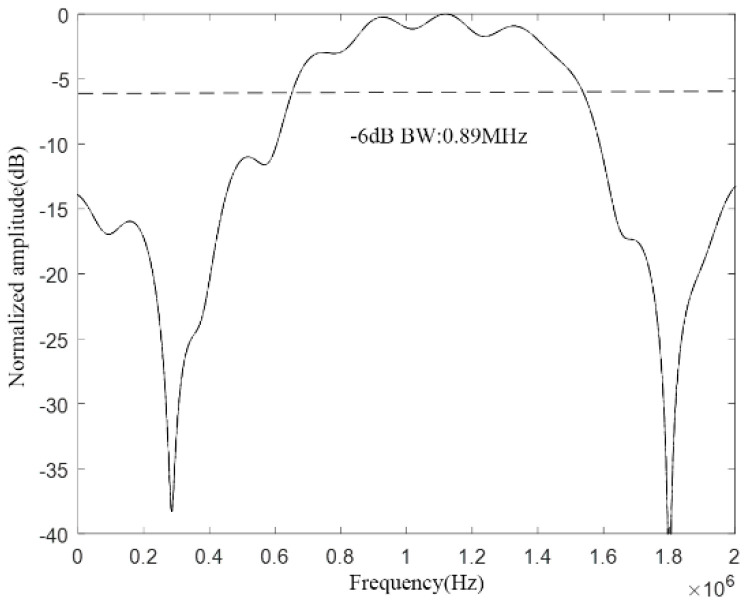
CMUT bandwidth in water.

According to the above analysis, the CMUT structure shown in Figure 20 is designed, and then the COMSOL finite element simulation software is used for modeling and analysis, and finally the bandwidth of the CMUT is obtained, as shown in Figure 21.

It can be seen from Figure 20 that the designed CMUT has a resonance frequency of 1.1 MHz in the water, −6 dB absolute bandwidth of 0.89 MHz, and its relative bandwidth of 81%, which meets the design requirements, which proves that the method proposed in this paper can be used in the water. The structural design of the wideband CMUT provides a reference.

## 5. Conclusions

In ultrasonic imaging, liquid flow measurement, distance measurement, ultrasonic identification, and other ultrasonic applications, wideband capacitive ultrasonic micromechanical transducer (CMUT) relies on its wide bandwidth, high sensitivity, low power consumption, high conversion efficiency, low cost and the advantages of easy processing, etc., having good application prospects. This article mainly describes the design method and characteristic analysis of the wideband CMUT. First, it introduces the influencing factors of the wideband CMUT cell and the CMUT bandwidth. Through simulation, it is found that the bandwidth of the CMUT cell increases as the radius–thickness ratio of its vibrating membrane increases, so the radius–thickness ratio of the membrane can be increased to obtain a wideband CMUT cell. The bandwidth of the CMUT array element increases with the decrease of its cell spacing, and it increases with the increase of the number of cells of different sizes. In order to improve the bandwidth characteristics of the CMT array element, it is necessary to reduce the cell spacing and increase the number of cells of different sizes. Next, a design method of wideband CMUT with hybrid cell structure is proposed, and CMUTs in air and water are respectively designed for simulation verification. The COMSOL simulation results show that the resonance frequency of the CMUT in the air domain is 3 MHz, and the relative bandwidth of −6 dB is 50%, while the resonance frequency of the CMUT in the water domain is 1.1 MHz, and the relative bandwidth of −6 dB is 81%, both of which realize the design of a wideband CMUT. In addition, the larger the frequency crossover range of adjacent cells, the flatter the frequency range of the CMUT array element and the better its bandwidth characteristics.

## Figures and Tables

**Table 1:** CMUT cell structure parameters.

Structure Unit	Used Material	Yang’s Modulus (GPa)	Poisson’s Ratio	Density (kg/m^3^)
Electrode	Al	70	0.350	2700
Membrane	Si	169	0.299	2332
Cavity	Vacuum	0	0	0
Insulation	SiO_2_	72	0.170	2200
Underlay	Si	169	0.299	2332

**Table 2:** CMUT cell structure parameters.

	Cell One		Cell Two		Cell Three	
**Structure Unit**	**Radius** **(μm)**	**Thickness** **(μm)**	**Radius** **(μm)**	**Thickness** **(μm)**	**Radius** **(μm)**	**Thickness** **(μm)**
Electrode	120	0.2	60	0.2	30	0.2
Membrane	240	41.6	120	10.4	60	2.6
Cavity	236	0.24	116	0.24	56	0.24
Insulation	240	0.1	120	0.1	60	0.1
Underlay	240	0.4	120	0.4	60	0.4

**Table 3:** Cell parameters of the three cell CMUT arrays.

	Cell One		Cell Two		Cell Three	
**Structure Unit**	**Radius** **(μm)**	**Thickness** **(μm)**	**Radius** **(μm)**	**Thickness** **(μm)**	**Radius** **(μm)**	**Thickness** **(μm)**
Electrode	29.5	0.2	30	0.2	30.5	0.2
Membrane	59	2.6	60	2.6	61	2.6
Cavity	55	0.24	56	0.24	57	0.24
Insulation	59	0.1	60	0.1	61	0.1
Underlay	59	0.4	60	0.4	61	0.4

**Table 4:** Cell parameters for the five cell CMUT array.

	Cell One		Cell Two		Cell Three		Cell Four		Cell Five	
**Structure Unit**	**Radius** **(μm)**	**Thickness** **(μm)**	**Radius** **(μm)**	**Thickness** **(μm)**	**Radius** **(μm)**	**Thickness** **(μm)**	**Radius** **(μm)**	**Thickness** **(μm)**	**Radius** **(μm)**	**Thickness** **(μm)**
Electrode	29	0.2	29.5	0.2	30	0.2	30.5	0.2	31	0.2
Membrane	58	2.6	59	2.6	60	2.6	61	2.6	62	2.6
Cavity	54	0.24	55	0.24	56	0.24	57	0.24	58	0.24
Insulation	58	0.1	59	0.1	60	0.1	61	0.1	62	0.1
Underlay	58	0.4	59	0.4	60	0.4	61	0.4	62	0.4

**Table 5:** The number of cells corresponding to different *w*.

***w***	20%	50%	80%
**The number of different cells required**	7	9	21
